# Patient’s perception of recovery after osteotome-mediated sinus floor elevation with Bio-Oss collagen compared with no grafting material: a randomized single-blinded controlled trial

**DOI:** 10.1186/s40729-021-00302-5

**Published:** 2021-03-22

**Authors:** Thomas Starch-Jensen, Niels Henrik Bruun

**Affiliations:** 1 Department of Oral and Maxillofacial Surgery, Aalborg University Hospital, 18-22 Hobrovej, DK-9000 Aalborg, Denmark; 2 Department of Clinical Medicine, The Faculty of Medicine, Aalborg University, Aalborg, Denmark; 3 Unit of Clinical Biostatistics, Aalborg University Hospital, Aalborg, Denmark

**Keywords:** Alveolar ridge augmentation, Dental implants, Health care surveys, Maxilla, Quality of life, Sinus floor augmentation

## Abstract

**Background:**

Osteotome-mediated sinus floor elevation with or without a grafting material is associated with high implant survival, intrasinus bone gain, and low frequency of complications. However, patient’s perception of recovery and satisfaction with the surgical intervention are rarely reported. The objective of the present randomized controlled trial was to assess patient’s perception of recovery after osteotome-mediated sinus floor elevation with Bio-Oss collagen compared with no grafting material. Forty healthy patients were randomly allocated to Bio-Oss collagen or no grafting material. Oral health-related quality of life was assessed by Oral Health Impact Profile-14 at enrollment. Patient’s perception of recovery was assessed by self-administrated questionnaires and visual analog scale evaluating pain, social and working isolation, physical appearance, duration and quality of life alterations, eating and speaking ability, diet variations, and sleep impairment after 1 week and 1 month, respectively. Descriptive statistics was expressed as mean percentage with standard deviation. Correlation between impaired oral health-related quality of life, age, gender, and recovery were assessed by *T* test. Level of significance was 0.05.

**Results:**

Osteotome-mediated sinus floor elevation is associated with high patient satisfaction, limited postoperative discomfort, and willingness to undergo similar surgery. Influence on patient’s daily life activities seems to be minimal and limited to the first postoperative days. Most patients managed to return to work and their routine daily activities after 0-2 days. Impaired preoperative oral health-related quality of life, gender, or younger age seems not to predispose for delayed recovery. However, number of days with pain, eating difficulties, and sleep disturbances were significantly increased with Bio-Oss collagen compared with no grafting material (*P*<0.05).

**Conclusion:**

Patient’s discomfort seems to be minimal and limited to the first postoperative days following osteotome-mediated sinus floor elevation with or without a grafting material. Impaired preoperative oral health-related quality of life, gender, or younger age seems not to predispose for delayed recovery.

## Introduction

Osteotome-mediated sinus floor elevation (OMSFE) with or without a grafting material have demonstrated high implant survival, intrasinus bone gain, limited peri-implant marginal bone loss, and low frequency of complications, as documented in systematic reviews and meta-analyses [1–5]. Implant survival rate and percentage of peri-implant marginal bone loss are commonly used to define a successful implant treatment outcome. However, clinical parameters do not necessarily reflect patient’s expectations and satisfaction with the surgical intervention or the implant-supported restoration. Therefore, success of implant treatment should not only focus on objective criteria but also include patient’s perception of recovery and patient-reported outcome measures (PROM), which is in agreement with a newly published systematic review and ITI consensus report concluding that there is an urgent need for standardized reporting of PROM in the field of implant dentistry [6, 7].

Prosthetic rehabilitation of partial or totally edentulous patients with implants significantly improves oral health-related quality of life (OHRQL) [8]. Self-administered questionnaire has demonstrated high patient satisfaction, low patient discomfort, and willingness to undergo the same type of surgery after OMSFE with simultaneous implant placement [9–12]. However, patient’s perception of recovery and PROM are influenced by various factors, such as gender, age, OHRQL, psychosocial factors, the surgical intervention, post-surgical morbidity, and complications [13, 14]. Nevertheless, assessment of predisposing factors as well as relationship between OHRQL and patient’s perception of recovery using validated self-administrated questionnaires after OMSFE with simultaneous implant placement have never previously been conducted. Therefore, the objective of the present randomized controlled trial was to test the hypothesis of no difference in patient’s perception of recovery after OMSFE with Bio-Oss collagen compared with no grafting material using validated self-administrated questionnaires.

## Material and methods

The protocol was prepared in full accordance with guidelines for reporting randomized controlled studies (CONSORT) (http://www.consort-statement.org/). The study was approved by The North Denmark Region Committee on Health Research Ethics (Approval No: N-20180027). Patients were recruited by public invitation through Facebook or admitted to the Department of Oral and Maxillofacial Surgery, Aalborg University Hospital, Denmark, for implant placement in the posterior part of the maxilla. Candidates were screened for inclusion and exclusion criteria at enrollment (Table 1). The residual bone height in the posterior maxilla was estimated by cone beam computed tomography at enrollment. Included patients received written as well as verbal information about the study protocol and signed an informed consent form before initiating the study. OMSFE and implant placement was free for the patients, but they had to pay for the prosthetic solution themselves. A total of 40 patients with a missing posterior maxillary tooth were included and randomly allocated to (1) OMSFE with Bio-Oss collagen and simultaneous implant placement or (2) OMSFE with no grafting material and simultaneous implant placement. A computer-aided block randomization was used to allocate included patients into two groups of same size. Based on sample size calculation and assuming a 10% dropout rate, it was planned to enroll 20 patients for each treatment group, in order to detect a 15% difference between the two groups in long-term implant survival, with a power of 0.8 and a significance level equal to 0.05.

**Table 1:** Inclusion and exclusion criteria

**Inclusion criteria**	
• >20 years • Missing one posterior maxillary tooth for more than four months • Residual alveolar bone height of the maxillary alveolar ridge ≥6 mm and ≤10 mm • Width of the alveolar ridge ≥6.5 mm • Mandibular occluding teeth • Able to understand and sign the informed consent • Single tooth gaps as well as free ended prosthetic solutions	
**Exclusion criteria**	
• Contraindications to implant therapy • Full mouth plaque score >25% • Progressive marginal periodontitis • Acute infection in the area intended for implant placement • Parafunction, bruxism, or clenching • Psychiatric problems or unrealistic expectations • Heavy tobacco use define as >10 cigarettes per day • Current pregnancy at the time of recruitment • Physical handicaps that would interfere with the ability to perform adequate oral hygiene • Inability or unwillingness to regularly attend the scheduled follow-up visits	

### Surgical procedure

**Fig. 1:**
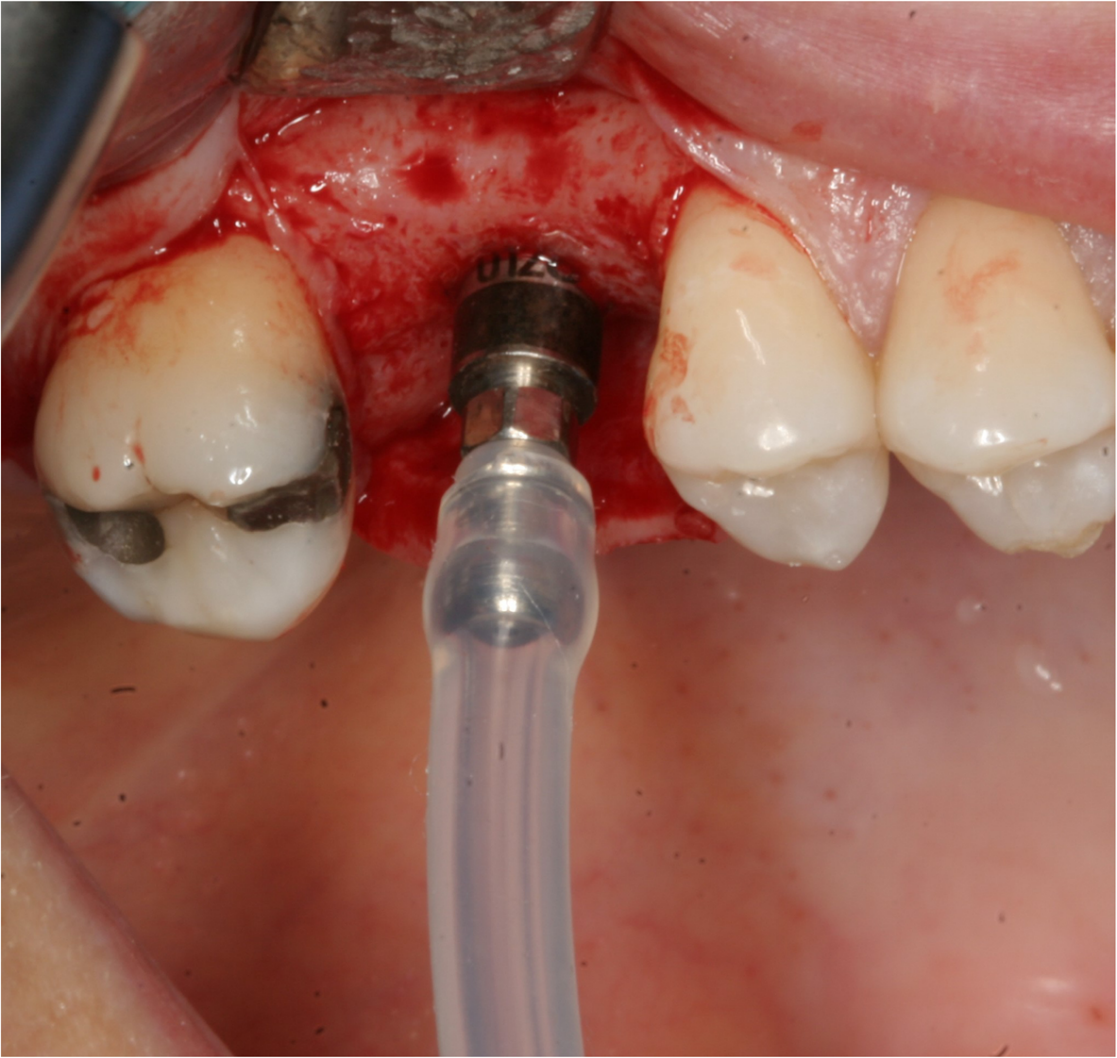
An watertight adaptor with a tube was inserted in the prepared implant bed and connected to a syringe containing physiological saline solution. The Schneiderian membrane was safely elevated by controlling the pressure of saline by means of the attached Physiolifter device

One hour prior to OMSFE, all patients were pre-medicated with analgesics involving 400 mg ibuprofen (Burana, Teva, Denmark) and 1000 mg paracetamol (Pamol, Takeda Pharma A/S, Denmark) and prophylactic antibiotic therapy including 2 g amoxicillin (Imadrax, Sandoz, Denmark) or clindamycin 600mg (Dalacin, Alternova, Denmark) if allergic to penicillin. All patients rinsed with 0.12% chlorhexidine solution for 1 min immediately before surgery. The surgical procedures were performed by the same trained surgeon (TSJ) in local anesthesia using lidocaine (2%) with 1:200,000 adrenaline (Xylocaine, Amgros I/S, Denmark). An intraoral marginal incision was performed at the implant site continuing into the gingival sulcus of the adjacent teeth. Mucoperiosteum was reflected exposing the alveolar process. An implant bed was successively prepared on the top of the alveolar crest according to the manufacturer’s drilling protocol. The depth of the drilling was ended at least 2 mm from the bottom of the maxillary sinus floor. The Schneiderian membrane including the original maxillary sinus floor was elevated to the planned implant length using calibrated osteotomes combined with piezosurgery and hydraulic pressure technique (Sinus physiolift II, Mectron, Carasco, Italy). A watertight adaptor with a tube was inserted in the prepared implant bed and connected to a syringe containing 2 ml of physiological saline solution (Fig. 1). The Schneiderian membrane was safely elevated by controlling the pressure of the liquid by means of the attached physiolifter device. The integrity of the Schneiderian membrane was checked by Valsalva maneuver and patients were asked, whether they had sensation of water in the nose or throat during use of the hydraulic technique. Moreover, the implant site was probed with the implant depth gage to feel the presence of an intact Schneiderian membrane. If the Schneiderian membrane was largely perforated with communication to the maxillary sinus, the patient was withdrawn from the study. A sealed randomization envelope was opened in order to allocate patients to (1) OMSFE with Bio-Oss collagen 250 mg (0.4–0.5 cm^3^, Geistlich Pharma AG, Wolhusen, Switzerland) and simultaneous implant placement (control group) or (2) OMSFE with no grafting material and simultaneous implant placement (test group). In the control group, the Bio-Oss collagen sponge was soaked in saline and pushed through each implant site underneath the Schneiderian membrane. A straight 13 mm implant (ASTRA TECH Implant System EV, diameter 3.6, 4.2 or 4.8, Dentsply Sirona Implants, Mölndal, Sweden) was inserted with a cover screw. Periosteum and mucosa were sutured with Vicryl 4-0 (Ethicon FS-2, Ethicon, St-Stevens-Woluwe, Belgium). No provisional restoration was inserted during the healing period. Patients were instructed to rinse with 0.12% chlorhexidine solution twice a day until suture removal has taken place after 7-10 days. Moreover, patients were instructed to avoid any physical activity that will abruptly raise or lower pressure in the sinus cavity as well as avoiding vigorous mouth rinsing, smoking, and touching the gums for at least 10 days following surgery. Postoperative analgesic was prescribed involving 400 mg ibuprofen, 1 tablet 3 times daily and 500mg paracetamol, 2 tablets 4 times per day, as long as required. All patients were prescribed postoperative antibiotics involving 800 mg phenoxymethylpenicillin (Primcillin, Meda, Denmark), 2 tablets 3 times daily for 7 days. In case of penicillin allergy, 300 mg clindamycin, 1 tablet 3 times daily for 7 days was used.

### Patient’s perception of recovery and patient-reported outcome measures

Oral Health Impact Profile-14 (OHIP-14) was used to assess OHRQL at enrollment. OHIP-14 is organized into seven conceptual dimensions including functional limitation, physical discomfort, psychological discomfort, physical disability, psychological disability, social disability, and handicap [14–16]. Two items are used to measure each dimension and consequently the questionnaire consists of 14 items. Response format of OHIP-14 was as follows: Very often = 4; fairly often or many times = 3; occasionally = 2; hardly ever or nearly never = 1; never/I do not know = 0. The OHIP-14 scale ranged from 0 to 56 and dimension score ranged from 0 to 8. The values of the 14 items and each dimension were summed to calculate the OHIP-14 severity score, with higher scores indicating poorer OHRQL.

Patient’s perception of recovery including pain, oral function impairments, general activity, and other symptoms was assessed after 1 week. A self-administrated questionnaire examined social isolation, working isolation, physical appearance, and mean duration of the quality of life alterations as well as questions whether they would undergo similar treatment again, if needed or if they would recommend this treatment to a friend or a relative, if indicated. Response format was yes or no. Eating ability and diet variations, speaking ability noticed, sleep impairment, and pain and discomfort at suture removal were also examined through self-administrated questionnaire after 1 week. Each item was evaluated by means of a four-point Likert-type rating scale. Response format was as follows: Not at all = 0; close to normal = 1; almost normal = 2; a little = 3. The rating score was calculated, with higher score indicating poorer patient recovery. Self-administrated questionnaire also examined how many days they have been on sick leave or been off work, had eating and speech difficulties, and how long their sleep and physical activity have been affected.

Patient’s perception of recovery was also examined by a self-administrated questionnaire after 1 month and supplemented by a 100 mm (0 = minimal to 100 = maximum) visual analog scale (VAS) assessing pain, social isolation, working isolation, eating ability, speaking ability, and sleep impairment.

Instructions for completing OHIP-14, self-administrated questionnaires and VAS were explained in detail to the patients. Patients completed the questionnaires by themselves, to prevent being influenced by the surgeons or nurses’ opinions and wills. Moreover, in order not to influence the compilation of the questionnaire, patients were not informed about their allocation group.

Intra-operative and postoperative complications including infection, wound dehiscence, nasal bleeding, explantation of implant or grafting material, exfoliation of grafting material or adverse events were also registered.

### Correlation of patient’s perception of recovery and oral health-related quality of life

Impaired OHRQL, gender, and age at enrollment were correlated to self-administrated questionnaires assessing patient’s perception of recovery after 1 week and 1 month. OHIP-14 item score of 10 or more was considered as impaired preoperative OHRQL.

### Statistical analyses

Data management and analysis was conducted using STATA (Data analysis and statistical software, version 16, StataCorp P, Texas, USA). Mean and standard deviations were reported when variables were considered continuous, e.g., scores and Likert scales. Comparisons for continuous variables were made by *t* test on the mean difference. Categorical variables were reported by counts and percentages. Dependencies for binary variables were tested by Fisher’s exact test. Dependencies for non-binary categorical were tested by chi-squared test of independence. Level of significance was 0.05. Sub-analysis where performed to analyze whether OHIP-14 levels were significantly different within each of the treatment modalities.

## Results

Forty patients underwent OMSFE with simultaneous implant placement. Patient characteristics are outlined in Table 2. There were no significant differences in distribution according to age, smokers and non-smokers, implant location, residual alveolar bone height, and width of the alveolar process. Significantly more females were included and allocated to OMSFE with Bio-Oss collagen (*P*=0.04). A minor perforation of the Schneiderian membrane was observed in one patient receiving OMSFE without a grafting material. Healing was uneventful in all patients and none of the included patients needed additional prescription of analgesics or antibiotic. No implant losses or graft infections were observed, but five patients described minor epistaxis during the first postoperative days. All patients attended postsurgical examinations and completed OHIP-14, self-administrated questionnaires, and VAS.

**Table 2:** Demographic characteristics of included patients

	OMSFE with Bio-Oss collagen	OMSFE without grafting material	***P*** value
Gender (male/female)	3/17	10/10	0.04*
Age at the time of OMSFE, mean (SD)	50.2 year (SD: 14.2)	48.1 year (SD: 9.1)	0.59
Smoking habits	0	1	1.00
Residual alveolar bone height (mm) at implant site, mean (SD)	6.8 (0.9)	7.2 (1.1)	0.36
Width of the alveolar ridge (mm) at implant site, mean (SD)	9.1 (0.6)	9.1 (0.8)	0.82
Implant location (second premolar)	9	5	
Implant location (first molar)	11	12	
Implant location (second molar)	0	3	
Number of implants with 3.6 mm diameter	1	0	
Number of implants with 4.2 mm diameter	7	5	
Number of implants with 4.8 mm Diameter	12	15	
Implant surface protruding into the sinus (mm), mean (SD)	6.2 (0.9)	5.9 (1.1)	0.18

*OMSFE* Osteotome-mediated sinus floor elevation; *SD* Standard deviation

*Statistically significant

**Fig. 2:**
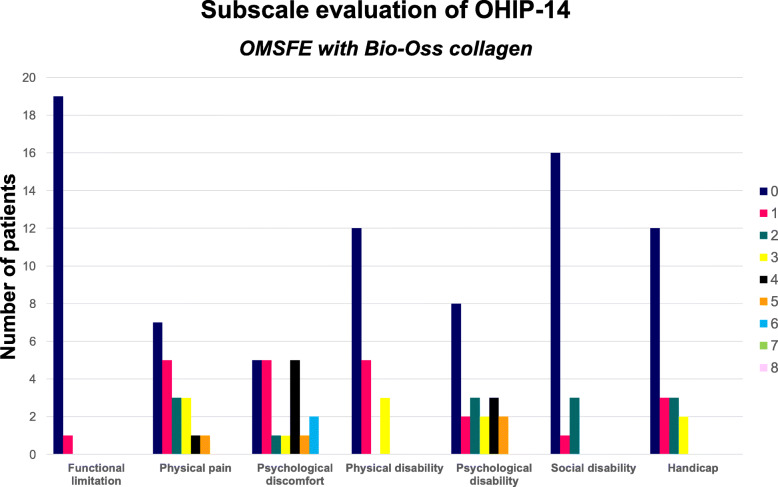
OHIP-14 subscale dimension score after OMSFE with Bio-Oss collagen

**Fig. 3:**
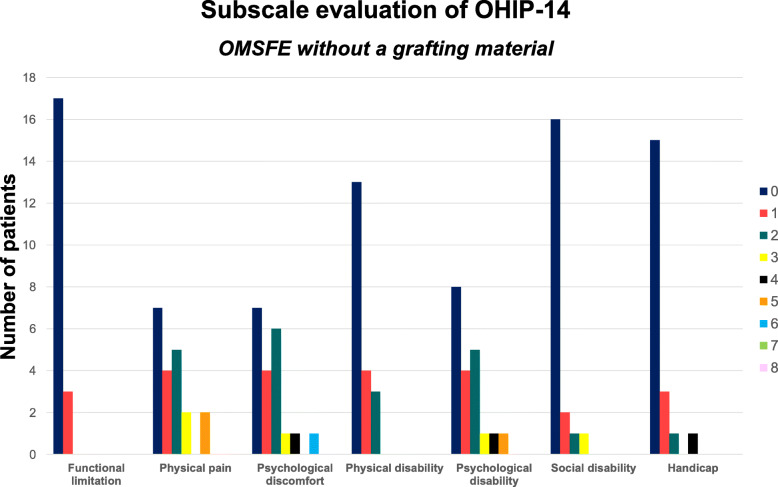
OHIP-14 subscale dimension score after OMSFE without a grafting material

Mean OHIP-14 and dimension score was 7.6 (SD: 9.5) and 0.6 (SD: 0.5) for OMSFE with Bio-Oss collagen compared with 6.0 (SD: 5.9) and 0.4 (SD: 0.3) without a grafting material, respectively, indicating no differences in OHRQL between the two groups (Table 3) (Figs. 2 and 3). Psychological discomfort and disability presented highest OHIP-14 dimension score, while functional limitation exhibited the lowest score indicating that self-consciousness, tension, and embarrassment were the factors which were significantly affected in both groups.

**Table 3:** Percentage distribution of responses to each question of OHIP-14 questionnaire

	Question	OMSFE with Bio-Oss collagen						OMSFE without grafting material					
		0	1	2	3	4	Mean	0	1	2	3	4	Mean
Functional limitation	Have you had trouble pronouncing any words because of problems with your teeth, mouth, or dentures?	95%	5%				0.05	90%	10%				0.1
	Have you felt that your sense of taste has worsened because of problems with your teeth, mouth, or dentures?	100%					0	95%	5%				0.05
Physical pain	Have you had painful aching in your mouth?	45%	45%	10%			0.65	55%	25%	15%	5%		0.7
	Have you found it uncomfortable to eat any foods because of problems with your teeth, mouth, or dentures?	50%	25%	20%	5%		0.8	45%	35%	15%	5%		0.8
Psychological discomfort	Have you been self-conscious because of your teeth, mouth, or dentures?	30%	20%	35%	10%	5%	1.4	50%	15%	30%	5%		0.9
	Have you felt tense because of problems with your teeth, mouth, or dentures?	55%	5%	30%	10%		0.95	65%	15%	5%			0.55
Physical disability	Has your diet been unsatisfactory because of problems with your teeth, mouth, or dentures?	80%	10%	10%			0.3	80%	15%	5%			0.25
	Have you had to interrupt meals because of problems with your teeth, mouth, or dentures?	70%	25%	5%			0.35	80%	15%	5%			0.25
Psychological disability	Have you found it difficult to relax because of problems with your teeth, mouth, or dentures?	75%	5%	20%			0.45	65%	20%	10%	5%		0.65
	Have you been a bit embarrassed because of problems with your teeth, mouth, or dentures?	35%	10%	25%	30%		1.5	50%	20%	30%			0.8
Social disability	Have you been a bit irritable with other people because of problems with your teeth, mouth, or dentures?	85%	5%	10%			0.25	85%	5%	10%			0.25
	Have you had difficulty doing your usual jobs because of problems with your teeth, mouth, or dentures?	95%		5%			0.1	90%	10%				0.1
Handicap	Have you felt that life in general was less satisfying because of problems with your teeth, mouth, or dentures?	55%	25%	15%	5%		0.7	75%	15%	10%			0.35
	Have you been totally unable to function because of problems with your teeth, mouth, or dentures?	95%		5%			0.2	95%		5%			0.2
		Total OHIP-14 score: 152 Mean OHIP-14 score for each patient: 7.7 Mean OHIP-14 score for all items: 0.6 (SD: 0.5)						Total OHIP-14 score: 109 Mean OHIP-14 score for each patient: 6.0 Mean OHIP-14 score for all items: 0.4 (SD: 0.3)					

*OMSFE* Osteotome-mediated sinus floor elevation

0 = never; 1 = hardly ever or nearly never; 2 = occasionally; 3 = fairly often or many times; 4 = very often

Questionnaire after 1 week revealed minor influence on patient’s daily life activities with both treatment modalities (Tables 4 and 5). All patients were satisfied with the treatment and would recommend it to friends and relatives (Table 4). However, number of days with eating difficulties and sleep disturbances were significantly increased after OMSFE with Bio-Oss collagen compared with no grafting material (*P*=0.04, *P*=0.02) (Table 6).

**Table 4:** Questionnaire assessing social and working isolation, physical appearance and quality of life alterations, at one week.

Question	OMSFE with Bio-Oss collagen		OMSFE without grafting material	
	Yes	No	Yes	No
**Social isolation**				
Did you keep your usual social activities?	95%	5%	100%	0%
Have you continued practicing your favorite sport or hobbies?	85%	15%	85%	15%
**Working isolation**				
Did you ask for sick leave or discontinue your work?	25%	75%	20%	80%
Did the surgery affect your performance at work?	10%	90%	5%	95%
Did anyone accompany you or drive you to work due to surgery?	0%	100%	0%	100%
Has this person discontinued his/her work to do so?	10%	90%	0%	100%
Did somebody accompany you for suture removal?	10%	90%	5%	95%
**Physical appearance**				
Have you noticed changes in your physical appearance?	25%	75%	15%	85%
Is it what you expected?	85%	15%	90%	10%
Has it been worse than expected?	15%	85%	5%	95%
Has it been better than expected?	80%	20%	95%	5%
**Mean duration of the quality of life alterations**				
Are you satisfied with the treatment?	100%	0%	100%	0%
Would you recommend it?	100%	0%	100%	0%
Would you repeat it?	95%	5%	100%	0%

*OMSFE* Osteotome-mediated sinus floor elevation

**Table 5:** Questionnaire assessing eating and speaking ability, diet variations, sleep impairment, pain, and discomfort at 1 week

Question	OMSFE with Bio-Oss collagen				OMSFE without grafting material			
	0	1	2	3	0	1	2	3
**Eating ability and diet variations**								
Did you continue with your usual diet?		80%	5%	15%		75%	20%	5%
Did you notice any change in the perception of taste?	75%	20%	5%		80%	20%		
Did you notice any change in chewing ability?	35%	20%	5%	40%	55%	15%		30%
Did you have problems opening your mouth?	70%	15%	5%	10%	75%	15%		10%
**Speaking ability noticed**								
Have you notice any change in voice?	80%	15%		5%	90%	10%		
Have you notice any change in your ability to speak?	75%	20%		5%	90%	10%		
When you talk with other people, do they understand you?	15%	85%			5%	95%		
**Sleep impairment**								
Have you had problems falling sleep?	65%	20%	5%	10%	75%	25%		
Have you experienced interruptions in sleep?	60%	15%		25%	85%	15%		
Have you felt drowsy?	15%	75%	5%	5%		100%		
**Pain and discomfort at suture removal**								
Has the removal of suture been uncomfortable?	95%			5%	75%	15%		10%
Has the appointment for suture removal caused you anxiety?	90%		5%	5%	70%	10%	5%	15%

*OMSFE* Osteotome-mediated sinus floor elevation

0 = not at all; 1 = close to normal; 2 = almost normal; 3 = a little

**Table 6:** Questionnaire assessing days of recovery at 1 week

Question	OMSFE with Bio-Oss collagen	OMSFE without grafting material	***P*** value
	Mean (range), SD	Mean (range), SD	
How many days have you been on sick leave or been off work?	0.6 (0-3), 1.0	0.3 (0-2), 0.6	0.19
How many days have you had eating difficulties?	2.1 (0-10), 2.7	0.7 (0-5), 1.2	0.04*
How many days have you had speech difficulties?	0.5 (0-7), 1.6	0.0 (0-0), 0.0	0.17
How many days has your sleep been affected?	1.0 (0-6), 1.9	0.0 (0-0), 0.0	0.02*
How many days has your physical activity been affected?	1.1 (0-4), 1.6	1.1 (0-14), 3.2	1.00

*OMSFE* Osteotome-mediated sinus floor elevation

*Statistically significant

Questionnaire after 1 month showed fast recovery with both treatment modalities (Table 7). However, the number of days with pain and sleep disturbances was significantly increased after OMSFE with Bio-Oss collagen compared with no grafting material (*P*=0.02).

**Table 7:** Questionnaire assessing pain, sick leave, performance, ability to eat, sleep, and speak at 1 month

Question	OMSFE with Bio-Oss collagen	OMSFE without grafting material	***P*** value
	Mean (range), SD	Mean (range), SD	
In how many days have you had pain after surgery?	3.2 (0-14), 3.3	1.3 (0-5), 1.3	0.02*
In how many days have you been on sick leave from daily activities such as work, school, etc. due to pain?	0.7 (0-5), 1.3	0.2 (0-5), 0.5	0.16
Did the operation affect your performance of your daily work? (VAS: 0-100)	19.9 (0-100), 34.2	8.1 (0-73), 18.0	0.18
In how many days have you been affected in your work?	0.9 (0-5), 1.4	0.3 (0-2), 0.6	0.07
Have you been able to eat a normal diet in the post-operative period? (VAS: 0-100)	68 (6-100), 35.2	84.2 (5-100), 26.2	0.11
In how many days have you been unable to eat your normal diet?	1.4 (0-5), 1.5	1.1 (0-5), 1.3	0.45
Have you noticed changes in your speech after surgery? (VAS: 0-100)	4.7 (0-48), 12.0	0.9 (0-6), 1.8	0.17
In how many days have you noticed changes in your speech?	0.6 (0-7), 1.6	0.0 (0-0), 0.0	0.11
Have you had trouble sleeping at night after surgery? (VAS: 0-100)	11.8 (0-100), 30.1	0.8 (0-6), 1.7	0.11
In how many days have your night’s sleep been affected?	0.9 (0-5), 1.7	0.0 (0-0), 0.0	0.02*

*OMSFE* Osteotome-mediated sinus floor elevation; *SD* standard deviation; *VAS* visual analog scale (0 = minimal to 100 = maximum)

*Statistically significant

There was no statistically significant correlation between impaired OHRQL (OHIP-14 score ≥10) at enrolment and patient’s perception of recovery after 1 week and 1 month (Table 8). Moreover, gender and younger age were generally not associated with impaired recovery. Though, males indicated significantly greater eating difficulties compared with females (*P*=0.03).

**Table 8:** Correlation between OHIP-14 item score at enrolment and patient’s perception of recovery after osteotome-mediated sinus floor evaluation

Question	OMSFE with Bio-Oss collagen			OMSFE without grafting material		
	OHIP-14 score < 10 (no.: 14) Mean (SD)	OHIP-14 score ≥ 10 (no.: 6) Mean (SD)	*P* value	OHIP-14 score < 10 (no.: 15) Mean (SD)	OHIP-14 score ≥ 10 (no.: 5) Mean (SD)	*P* value
**Week**						
How many days have you been on sick leave or been off work?	0.4 (0.9)	1.2 (1.2)	0.11	0.2 (0.6)	0.4 (0.5)	0.50
How many days have you had eating difficulties?	1.9 (2.0)	2.7 (4.1)	0.55	0.8 (1.4)	0.4 (0.5)	0.54
How many days have you had speech difficulties?	0.7 (1.9)	0.0 (0.0)	0.38	0.0 (0.0)	0.0 (0.0)	
How many days has your sleep been affected?	1.1 (2.1)	0.7 (1.2)	0.62	0.0 (0.0)	0.0 (0.0)	
How many days has your physical activity been affected?	1.2 (1.8)	0.7 (1.2)	0.50	1.4 (3.6)	0.0 (0.0)	0.41
**Month**						
How many days have you had pain after surgery?	2.9 (3.8)	3.8 (1.5)	0.59	1.3 (1.5)	1.0 (0.7)	0.64
In how many days have you been on sick leave from daily activities?	0.5 (1.4)	1.0 (1.1)	0.45	0.1 (0.5)	0.4 (0.5)	0.34
Did the operation affect your performance of your daily work? (VAS)	14.3 (33.9)	33.0 (33.9)	0.27	9.7 (20.6)	3.4 (3.7)	0.51
In how many days have you been affected in your work?	0.6 (1.4)	1.5 (1.4)	0.23	0.2 (0.6)	0.4 (0.5)	0.50
In how many days have you been unable to eat your normal diet?	1.6 (1.4)	1.2 (1.9)	0.60	1.3 (1.5)	0.6 (0.5)	0.35
Have you been able to eat a normal diet in the post-operative period? (VAS)	59.1 (38.0)	89.0 (14.1)	0.08	86.9 (21.1)	76.2 (40.0)	0.45
Have you noticed changes in your speech after surgery? (VAS)	6.4 (14.2)	0.7 (0.8)	0.34	0.7 (1.7)	1.6 (2.3)	0.34
In how many days have you noticed changes in your speech?	0.9 (1.9)	0.0 (0.0)	0.29	0.0 (0.0)	0.0 (0.0)	
Have you had trouble sleeping at night after surgery? (VAS)	15.2 (35.5)	4.0 (7.1)	0.46	0.5 (1.2)	1.8 (2.7)	0.15
In how many days have your night's sleep been affected?	1.0 (1.8)	0.7 (1.2)	0.69	0.0 (0.0)	0.0 (0.0)	

*OMSFE* Osteotome-mediated sinus floor elevation; *SD* Standard deviation; *VAS* Visual analog scale (0 = minimal to 100 = maximum)

## Discussion

The present study demonstrates no significant differences in patient’s perception of recovery and OHRQL after OMSFE with Bio-Oss collagen compared with no grafting material as evaluated by OHIP-14, self-administrated questionnaires, and VAS. High patient satisfaction, minimal discomfort, willingness to undergo the same type of surgery, and low frequency of intra- and postoperative complications was observed with both treatment modalities. The influence on daily life activities including pain, minor swelling, and sick leave from work seems to be minimal and limited to the first postoperative days and most patients managed to return to work and their routine daily activities after 0-2 days. There were no obvious differences in social and working isolation, physical appearance, quality of life alterations, speaking ability, and discomfort between the two treatment modalities. However, the number of days with pain, eating difficulties and sleep disturbances was significantly increased after OMSFE with Bio-Oss collagen compared with no grafting material, although the difference seems to have limited clinical relevance.

The present study is characterized by following limitations including small patient sample, solely collecting postsurgical information corresponding to 1 week and 1 month, few self-administrated questionnaires, and no registration of quantity and period of need for analgesics after OMSFE. Moreover, association between socioeconomic status, educational background, monthly income, level of daily physical functioning, and patient’s perception of recovery was not examined. Conclusions drawn from the results of this study should therefore be interpreted with caution.

OHRQL questionnaires provide valid and reliable information about the efficiency of treatment methods as well as physical, psychological, and social consequences for patients with different health states. OHIP-14 and OHIP-49 are the most commonly used questionnaire designed to measure impairment of OHRQL and patient’s perception of the social impact of oral disorders on their well-being [15, 16]. However, OHIP states the patient’s overall oral impairment, and does not take a specific surgical intervention into account. Consequently, OHIP is frequently used in combination with additional OHRQL questionnaires to interpret patient’s perception of a specified treatment modality and recovery.

Patient-related predictors for delayed clinical recovery have important clinical implications for the decision-making process before initiating prosthetic rehabilitation of the atrophic posterior maxilla with implants. A previous study assessing patient perception of recovery after maxillary sinus floor augmentation concluded that female and younger age were patient-related predictors for delayed recovery [13]. In the present study, preoperative OHIP-14 questionnaire was combined with self-administrated questionnaires to assess patient’s perception of recovery after OMSFE disclosing no correlation between impaired preoperative OHRQL and worsened perception of recovery. Moreover, no association between gender or younger age and impaired recovery were identified.

OHRQL and PROM reflects psychosocial parameters related to patient’s perception of the surgical intervention and recovery including pain, swelling, discomfort when eating, sleeping, working, and social interaction as well as their self-esteem and satisfaction with their oral health. However, OHRQL and patient’s subjective assessment of the treatment and recovery after OMSFE is seldom reported [9–12]. A previous study assessing patient’s perception of the surgical procedure after OMSFE with or without a grafting material revealed that more than 90% of the patients were satisfied with the implant therapy and would undergo similar therapy again if necessary as well as recommend the treatment to a friend or relative, if indicated [9]. However, approximately 23% of the patients found the surgical experience unpleasant and 5% of the patients experienced vertigo, nausea, and felt disoriented after OMSFE. Moreover, five patients experienced psychological problems, which needed medical assistance [9]. In the present study, all patients were satisfied with the implant therapy and would recommend the treatment to a friend or relative. Only one patient would not undergo similar therapy again. None of the patients reported disorientation, nausea, or vertigo after OMSFE, but discomfort during osteotome hammering was frequently described. Benign paroxysmal positional vertigo has previously been reported after OMSFE with osteotome hammering due to detachment of otoliths from the otoconia layer of the utricular macula [17–19]. Although OMSFE induced benign paroxysmal positional vertigo is rare, the experience of postoperative disorientation and vertigo will certainly affect patient’s perception of recovery.

Patient preferences have previously been assessed after OMSFE with Cosci technique using a series of atraumatic lifting drills compared with Summers’ technique including flat-tipped osteotomes [10]. No discomfort or complications was reported after Cosci technique, whereas postoperative swelling or headache was described in 80% of the patients with the Summers’ technique [10]. In the present study, sinus membrane elevation was performed with Summers’ technique in combination with hydraulic technique. None of the patients complained of postoperative headache. Previous studies assessing OMSFE with hydraulic pressure and vibrations have demonstrated high implant survival rate, limited peri-implant marginal bone loss, and minimal patient discomfort [20–22]. Moreover, no pain was reported in 67% of the patients and 56% experienced no swelling after hydraulic OMSFE [22].

PROM after OMSFE with simultaneous implant placement compared with implant placement in native bone have demonstrated no significant difference in discomfort, willingness to undergo the same type of surgery, pain, and use of postoperative analgesics [11]. Both treatment modalities revealed low postoperative pain and medication [11]. These results are in agreement with the present study.

OMSFE with simultaneous implant placement has demonstrated a significant higher level of pain score compared with maxillary sinus floor augmentation on the day of surgery [12]. Level of pain was assessed by VAS revealing a significant decreased in pain from the first postoperative day with both treatment modalities. OMSFE was characterized by significantly lower incidence of swelling, bruising, and nasal discharge/bleeding as well as less severe limitation in swallowing, continuing daily activities, eating, speaking, opening the mouth, and going to school/work compared with maxillary sinus floor augmentation [12]. In the present study, most patients reported only minor pain during the first postoperative day and minimal swelling and discomfort.

Schneiderian membrane perforation is the most frequent intraoperative complication after OMSFE, with prevalence up to 40% [1–3, 23]. Perforation of the Schneiderian membrane may result in communication and displacement of grafting material into the maxillary sinus. Previous studies assessing Schneiderian membrane perforation after OMSFE using ultrasonic piezoelectric vibration and hydraulic pressure have reported an incidence of 3-9% [20, 24]. In the present study, Schneiderian membrane perforation occurred 2.5%. Perforation of the Schneiderian membrane after OMSFE may not necessarily influence patient’s perception of recovery but increase the probability of postoperative infection, sinusitis, and subsequently failure of the implant [25].

## Conclusions

Within the limitations of the present study, it can be concluded that OMSFE with Bio-Oss collagen compared with no grafting material is associated with high patient satisfaction, minimal discomfort, willingness to undergo the same type of surgery, and low frequency of intra- and postoperative complications. The present study demonstrates that 2-3 days of recovery from OMSFE is usually needed for patients to resume oral function and daily activities without limitations or need for analgesics. Moreover, impaired preoperative oral health-related quality of life, gender, or younger age seems not to predispose for delayed recovery.

## Data Availability

Study protocol and all data are available from the corresponding author on reasonable request.

## Abbreviations

OHIP: Oral health impact profile
OHRQL: Oral health-related quality of life
OMSFE: Osteotome-mediated sinus floor elevation
PROM: Patient-reported outcome measures
SD: Standard deviation
VAS: Visual analog scale
